# Molecular determinants of outcomes in meningiomas

**DOI:** 10.3389/fonc.2022.962702

**Published:** 2022-08-12

**Authors:** John Lynes, Gabriel Flores-Milan, Sebastian Rubino, John Arrington, Robert Macaulay, James K. C. Liu, Andre Beer-Furlan, Nam D. Tran, Michael A. Vogelbaum, Arnold B. Etame

**Affiliations:** ^1^ Division of Neurosurgery, Moffitt Cancer Center, Tampa, FL, United States; ^2^ Department of Neuro-Oncology, Moffitt Cancer Center, Tampa, FL, United States; ^3^ Department of Radiology, Moffitt Cancer Center, Tampa, FL, United States; ^4^ Department of Pathology, Moffitt Cancer Center, Tampa, FL, United States

**Keywords:** meningiomas, genetics, epigenetics, outcomes, targeted therapy, molecular classification, brain tumor

## Abstract

Meningiomas are the most common intracranial primary tumor in adults. Surgery is the predominant therapeutic modality for symptomatic meningiomas. Although the majority of meningiomas are benign, there exists a subset of meningiomas that are clinically aggressive. Recent advances in genetics and epigenetics have uncovered molecular alterations that drive tumor meningioma biology with prognostic and therapeutic implications. In this review, we will discuss the advances on molecular determinants of therapeutic response in meningiomas to date and discuss findings of targeted therapies in meningiomas.

## Introduction

Meningiomas are the most common primary intracranial benign tumor in adults. They commonly present due to seizures, focal neurologic deficit, or symptoms of elevated intracranial pressure such as headaches or nausea (1). They account for 39% of all tumors and 54.5% of all non-malignant primary intracranial tumors, with a median age of diagnosis of 66 years old. Incidence in the United States from 2014-2018 was 9.49/100,000, with a 2.3 higher incidence in women and more common among non-Caucasian populations (2). Approximately 36,130 patients were diagnosed with meningiomas in 2021 in the United States alone. A large majority of meningiomas are benign with 80% being grade 1, 18.3% grade 2 or atypical, and 1.3% grade 3 or malignant. Prognosis of patients with meningioma correlates with tumor grade. In non-malignant meningiomas, overall 5-year survival of 88.2%, and 10-year survival of 83.7%, while 5-year survival of malignant meningiomas is 67.5% (3). Meningiomas have been historically reported in 4.6% of patients over 80 years old having one meningioma and 8.2% having multiple at time of autopsy (4). However, a recent review evaluating incidental radiographic discovery of meningiomas found an overall rate of 0.52% in the general population (5). Most meningiomas are asymptomatic at time of presentation (6).

While a majority of meningiomas are sporadic, a subset of cases is associated with familial syndromes. Neurofibromatosis type 2 (NF2) is the most common of these hereditary syndromes, but increased risk of meningioma formation also occurs in multiple endocrine neoplasia (MEN) type 1, von Hippel-Lindau (VHL), Li-Fraumeni syndrome, Gorlin syndrome, Cowden syndrome, nevoid basal cell carcinoma syndrome, BAP1 tumor predisposition syndrome, Rubinstein-Taybi Syndrome, and familial meningiomatosis (7). Through identification of this predisposition and subsequent investigation of the genetic alterations within these syndromes, we hope to contribute to a greater understanding of pathogenesis of the sporadic disease as well.

## Radiographic features predictive of grade and outcomes

While meningiomas can have a variable appearance on computerized tomography (CT) and magnetic resonance (MR) imaging, there are characteristic imaging features that allow for a confident and accurate imaging diagnosis. Classically, meningiomas are seen as avidly enhancing and sharply marginated extra-axial masses with a broad based dural attachment and associated smooth dural enhancement (the dural “tail”). There may be adjacent dural involvement with increased enhancement, nodularity, or thickening compared to uninvolved dura. Meningiomas are typically isointense to grey matter on T1 weighted (T1W) and T2 weighted (T2W) MR imaging as well as being isodense to grey matter on non-contrast CT (NCCT) imaging. Meningiomas may have calcifications which are seen best on NCCT and on susceptibility weighted imaging (SWI) MR images. Hyperostosis of the adjacent calvarium is a common imaging finding which is also seen best on NCCT and can be seen with both reactive osseous changes as well as calvarial invasion by meningioma. While these tumors are most often associated with dural structures, they also arise less frequently within the ventricles or optic nerve sheath. As meningiomas grow, they can displace and compress the adjacent brain leading to a CSF cleft seen between meningioma and brain seen best on T2W MR imaging. Meningiomas can invade the underlying brain. Adjacent brain vasogenic edema is also a common imaging finding and can be seen both with and without brain invasion.

Given the variability of radiographic appearance of meningiomas and wide range of pathologies that may involve the dura including inflammatory and infectious etiologies, as well as, hematologic and metastatic malignancies, additional imaging strategies are valuable to assist in accurate diagnosis. More effective imaging technologies are needed to help diagnose various types of meningiomas, as well as, to distinguish meningioma grades. While MR perfusion may help distinguish between meningiomas from some dural-based metastases, there are no MR perfusion findings that are diagnostic for meningiomas which have been shown to have similar hyperperfusion to Merkel cell carcinoma, renal cell carcinoma, and melanoma (8).

Attempting to radiographically determine meningioma grade remains a persistent challenge. Lee EJ et al. reported from 232 patient that only 25.4% showed rapid growth in 5-year interval follow-up (9). In addition, the authors showed that tumor size, absence of calcification, peritumoral edema and hyperintense or isointense signal on T2-weighted MRI were predictors of tumor growth (9). Studies have reported that heterogeneous enhancement, lack of distinct space separating tumor from adjacent brain (10), increased hyperintensity on apparent diffusion coefficient (ADC) (11, 12), and differential activity on O-(2-[18F]fluoroethyl-)-L-tyrosine (F-FET) positron emission tomography have been reported to be associated with higher histologic grade (13). Given the complexity of often subtle differences in radiographic appearance, groups have utilized radiomics and machine learning in an effort to provide better predictive models for meningioma subtyping. However, these studies are limited by the methodology of machine learning, particularly the single or limited-center retrospective patient populations that limit generalizability (14). Gallium-68-DOTATATE PET/CT which targets the somatostatin receptor 2 (SSTR2) has shown utility in meningioma radiographic diagnosis and distinguishing from other pathologies (15). A study found that increased uptake by DOTATE PET/CT is correlated with increased growth rate in grade 1 and 2 meningiomas, though prognosis could not be made grade 3 meningiomas (16). Furthermore, DOTATATE PET/CT allows for a greater delineation between meningioma and other adjacent physiologically contrast enhancing structures, such as pituitary tissue or venous sinuses, as well as post treatment effects (17). The combination of MRI and DOTATATE PET/CT can also aid surgical planning with the goal of maximizing extent of resection as well as improving radiation therapy target planning (18). Beyond diagnosis and grading of meningiomas, the standardization of nomenclature for assessing radiographic response of a known meningioma to treatment has also been described by the Response Assessment in Neuro-Oncology (RANO) working group as complete response (resolution of lesions for at least 8 weeks), partial response (>50% decrease from baseline), minor response (25-50% decrease), progressive disease (>25% growth from baseline), and stable disease (19). These categories allow for more effective reporting and comparison of groups reporting results in effort to determine novel or improved treatment strategies.

## Histopathologic features and current classification

While advances in imaging technique and technology may lead to more effective diagnosis in the future, the gold standard for diagnosis remains tissue analysis. Histologically, meningiomas are typically characterized on H&E staining by whorls of cells with nuclear pseudo-inclusions, pseudo-syncytial growth, and psammoma bodies representing circular calcifications. Additionally, immunohistologic staining positivity for Somatostatin Receptor 2a (SSTR2a) is diagnostic (20). The World Health Organization (WHO) classification scheme for central nervous system tumors categorizes meningiomas into 15 subtypes. Thereafter, the 2016 update added histologic evidence of brain invasion with mitotic count greater than 4, or having 3 of 5 features: necrosis, loss of whirling or fascicular architecture, prominent nucleoli, high cellularity, and cells with high nuclear to cytoplasmic ratio as being diagnostic of atypical, grade 2, meningioma (21). However, reproducibility of grading remained a challenge with one study reporting only 87.2% agreement of meningioma grade between different observers involved in a multicenter trial (22). The 2021 WHO classification clarified several historically used diagnostic criteria. Previously, choroid and clear cell meningioma subtypes were classified as grade 2, while rhabdoid and papillary subtypes were classified as grade 3. However, the authors indicate that though meningiomas with these histologic appearances largely fall within those grades, that appearance alone should not determine the grade, but rather by the grading criteria introduced in 2016. The 2021 update additionally reports commonly altered genes in meningiomas, but largely does not use them as grading criteria with the exception of CDKN2A/B homozygous deletion and TERT promoter mutation as diagnostic for grade 3 meningiomas (23).

The introduction of genetic alterations into meningioma grading reflects a growing body of evidence for the utility of greater understanding of genetic and molecular profiling in meningiomas.

## Molecular features in meningiomas

Historically, clinicians have relied only on histological features for classification into three pathological grades. In 2021, the World Health Organization (WHO) Classification of the Central Nervous System (CNS) newly included a series of molecular biomarkers. For the purpose of grading, the classification scheme lists SMARCE1 (clear cell subtype), BAP1 (rhabdoid and papillary subtypes), and KLF4/TRAF7 (secretory subtype) mutations. Separate from grading, TERT promoter mutation, homozygous deletion of CDKN2A/B, and loss of nuclear H3K27me3 expression were included as being indicators of poor prognosis (23).

A genomic study of 300 meningiomas showed mutations in *TRAF7* in approximately 25% of all meningiomas, AKT1 mutations in 10-15% (affecting the PI3K signaling pathway) and KLF4 mutations in 10%. SMO mutations which activate Hedgehog signaling were identified in 5% of non-*NF2* mutant meningiomas and *NF2* and/or chromosome 22 loss were more likely to be atypical meningiomas (24). Furthermore, such mutations were correlated to anatomical tumor location and traditional histological analysis (24, 25). SMARCE1 heterozygous loss of function mutation has been associated with spinal meningiomas and in tumors with clear-cell histology (26). TERT promoter mutation was found to be key in meningiomas undergoing malignant histological progression and predictor for poor survival (27–29).

The impact of molecular profiling of meningioma has been studied and validated to predict clinical courses that affect the post-operative management including imaging surveillance and need for adjuvant radiation (30–33). WHO grades II and III meningiomas have aggressive clinical courses, although poor outcomes may occur in a subset of low-grade lesions. Youngblood et al. found that low grade meningiomas harboring a particularly genomic group (Hedgehog, NF2, PI3K and TRAF7) recurred at rate 21.9 times higher and 17.2 times higher than would be expected given their more benign histopathology (30). Patel et al. analyze 160 meningiomas by classifying in 3 groups base on molecular profile and found increased expression of FOXM1 and MYBL2 causing DREAM complex loss of its repressive activity associated with recurrence (31). This genomic event represented aggressive tumor behavior, and 79% of a sub-group tumors showed a genomic loss of both 1p and 22q (31).

A large cohort retrospective study attempted an integrated scoring system that included histology and molecular risk stratification proving higher accuracy in clinical outcomes, including stratification by DNA methylation (32). Vasudevan et al. found FOXM1 targets accounted for 11% of genes enriched in WHO grade III meningiomas, compared with only 3% of genes in WHO grade I meningiomas, correlating this gene to poor clinical outcomes (33). Furthermore, Magill et al. analyze intratumor heterogeneity suggesting that the loss of chromosome 22q is an early event that tumor evolution, but prove spatially distinct patterns of FOXM1, CDH2, and PTPRZ1 expression providing understanding why meningiomas grow asymmetrically (34).

There is limited data from *in vivo* models to identify key drivers of meningioma cell invasion that may play a role in the mechanism of recurrences, prognostication, and potential targets for therapies of high grade meningiomas. Erson-Omay et al. recently demonstrated that sporadic multiple meningiomas in the same patient can show both genomic and histologic heterogeneity (35). These tumors can have both mono- and multi-clonal origin which can be observed in both NF2-loss and non-NF2 mutant tumors. In addition, those monoclonal multiple meningiomas can acquire inter-tumor heterogeneity due to additional somatic alterations through branched evolution (35). Nigim et al. analyzed the expression of β1 integrin of clinical meningioma specimens and found *in vivo* murine model utilizing two patient-derived high grade meningioma xenografts, that antibody therapy targeting β1 integrin decreased high grade meningioma cells proliferation and extended overall survival (36). A preclinical study found that 9% of 108 meningiomas demonstrated mutations in the PI3K/AKT/mTOR pathway, suggesting it may play an important role in the growth of meningiomas (37). However, a phase II trial and two retrospective studies failed to show efficacy of bevacizumab and everolimus in patients with recurrent high grade meningiomas (38–40). Appears to be that genetic profiling of meningioma in the next decade will provide prognostication in risk profile stratification for recurrence, risk of malignant progression or transformation and potentially improve efficacy of current target therapies.

## Molecular classification schemes for meningiomas

Meningiomas have historically been classified based on histological appearance into 15 histologic subtypes. However, with advances in genetic and epigenetic underpinnings of meningioma pathogenesis several molecular classification schemes have been described with the intent of developing clinically relevant tools.

### Classification based on genetic alterations

Approximately 80% of sporadic meningiomas harbor mutations in one of seven genes or pathways, prompting a potential classification scheme on that basis (**Table 1**). The seven subgroups are 1) Ne*urofibromitosis-2* (NF2) with or without SMARCB1, 2)*TNF Receptor-Associated Factor* 7 (TRAF7) alone, 3)TRAF7 with *Kruppel-Like Factor 4* (KLF4), 4) phosphoinositide 3-kinase (PI3K) pathway including PIK3CA, PIK3R11, and AKT1, 5) Hedgehog (HH) pathway including SMO, SUFU, PRKARIA, 6) RNA Polymerase II Subunit A (PPOL-R2A), and 7) SMARCE1, with over 50% of tumors being within the first subgroup, NF2 mutations (41). There are multiple observed patterns supporting this classification system and its clinical relevance. Meningiomas in different anatomic locations reliably follow these subgroups. For example, HH pathway mutations all localize to the midline anterior skull base, while NF2 plus SMARCB1 mutated tumors involve the falx (42). BAP1 mutant meningiomas localize to cerebral convexities, while SMO mutant meningiomas are located in the anterior skull base, but not midline. Posterior fossa meningiomas harbor mutations in NF-2, POLR2A, or AKT1E17K (43).

Furthermore, the different subgroups may have different epidemiology and clinical behavior. NF2 mutant tumors are often larger, atypical with a more aggressive clinical course, associated with preoperative seizures, and found in a greater proportion of male patients (44) (42). Among the six non-NF2 mutant subtypes, the TRAF7 mutated alone subtype is the most common in 25% of sporadic meningiomas (41) and is associated with a higher grade (42). Conversely, the HH pathway altered tumors are associated with less aggressive clinical behavior (45). By developing genetic classification systems, there may be benefit in the ability to identify patients that have greatest potential benefit from targeted therapies.

**Table 1:** Meningioma classification based on genetic mutations.

Genetic Mutation Subgroup	Clinical features
NF2	Most common, larger, more aggressive course. Highest seizure risk. Male predominance (1). Falcotentorial location (2).
TRAF7	Second most common. associated with higher grade. Higher likelihood of hyperostosis (1) Midline and lateral skull base location (2).
TRAF7 with KLF4	High peritumor edema (1). Midline and lateral skull base location (2).
PI3K Pathway	Low recurrence risk (3) Midline and lateral skull base location (2).
HH pathway	Less aggressive, low grade (1). Localize to midline anterior skull base (2).
PPOL-R2A	Benign, female predominance. Sella, clivus, and posterior fossa location (2).
SMARCE1	High recurrence risk (1), faster growth. Lateral skull base, anterior falcine location (2).

### Classification based on epigenetic factors

In addition to genetic mutations and alterations, patterns of epigenetic alterations have also been discovered. An evaluation of the transcriptome of 160 meningiomas including all grades and subsequent clustering analysis identified three molecular subtypes of meningiomas (**Table 2**). When this analysis was then applied to other databases, the three subtypes predicted progression free survival more accurately than traditional WHO grading, as well as PFS of tumors of different molecular subtypes within each WHO grade (31).

**Table 2:** Meningioma classification based on transcriptome analysis.

Transcriptome Subtypes	Features
Type A	Low proliferation index. Anterior skull base. 2:1 female to male. No significant chromosomal changes. Only type with TRAF7. High prevalence of KLF4 and AKT1. No NF2 mutations.
Type B	Intermediate proliferation index. 2:1 female to male. Significant prevalence of loss of chr22q. NF2 mutations. Highest prevalence of SMARCB1.
Type C	High proliferation index. Falcine, occipital regions. 56% male. Significantly shorter PFS than Type A or B irrespective of WHO grade. In addition to NF2, numerous chromosomal abnormalities.

Furthermore, a retrospective DNA methylation analysis was performed on 479 patients to identify six methylation classes, which was then compared to genetic mutations and RNA sequencing findings (46). The investigators reported that while the system of six classes based on DNA methylation did have significant and consistent overlap with particular histologic or genetic subtypes, when they did not match, the clinical behavior was better predicted by methylation class. For example, a tumor graded as atypical but harboring a methylation classification typically seen in benign meningiomas would behave as a grade 1 tumor as well as the reverse (46). These findings suggest that by having classification systems that can more accurately predict tumor behavior, clinicians can more effectively make treatment decisions such as the use of more aggressive therapies versus observation. These findings were corroborated by a recent study utilizing DNA methylation profiling of over 500 meningiomas to project clinical outcomes based on categorization into three subtypes. The authors found that Merlin-intact (NF2 wild-type) subtype have the best outcome, followed by the immune enriched, and hypermitotic subtypes. The hypermetabolic subtype was associated with CDKN2A/B hypermethylation and NF2 loss, and a majority of these tumors are grade 2 or 3 (47).

### Integration of molecular alterations

Due to the complexity of differing classification schemes, Nassiri et al. (48) developed an integrated system with four groups, which they designate molecular groups 1 through 4 (MG1-MG4). The molecular groups were determined by integrating clustering found in DNA methylation and mRNA abundance clusters. Histologic grades were spread between molecular groups with MG1 containing grade 1 and grade 2 meningiomas, while MG2 through MG4 contained all grades. However, grade 2 and 3 tumors were increased in MG3 and MG4. When correlated with clinical behavior of these tumors, MG3 and 4 tumors were found to have lower progression-free survival than MG1 and 2 tumors irrespective of histologic grade. Almost all MG1 tumors contained NF2 mutations, while mutations in TRAF7, AKT1, KLF4, and POLR2A were found only in MG2 tumors. MG3 and MG4 tumors had significantly enriched mutations in epigenetic regulatory genes and tumor suppressor genes compared to MG1 and 2. MG1 tumors were typically diploid, though with chromosome 22q loss corresponding to NF2 loss. MG2 tumors fell into two categories: copy number neutral with point mutations, and lack of point mutations but with corresponding chromosomal polysomies on locations for TRAF7, AKT1, KLF4, or SMO. MG3 and MG4 tumors had high levels of aneuploidy and interchromosomal fusions. Some MG4 tumors additionally demonstrated gain of chromosome 1q and loss of chromosome 10. These alterations and further proteomics to investigate resulting changes to gene products led the group to attribute characteristics to the subgroups. MG1 tumors were immunogenic, MG2 tumors were benign NF2 wild-type, MG3 were hypermetabolic, and MG4 were highly proliferative (48).

## Current treatment modalities

Although the overwhelming majority of meningiomas are histologically benign, meningiomas can present as a significant source of physical and psychological morbidity in patients. Even in absence of symptoms that may affect a patient’s functional status, health-related quality of life (HRQOL) scores are decreased in patients secondary to awareness of an intracranial tumor and subsequent psychological distress from anxiety and depression (49). Neurocognitive and neurological symptoms resulting in physical limitations from symptomatic meningiomas further impair quality of life. Treatment of the lesion by surgery or radiation therapy as appropriate, conversely, improves HRQOL scores from pre-treatment baseline, though it may not improve to the level of the general population without this disease (49). As such, effective treatment of meningiomas and minimization of deleterious treatment sequelae provides benefit to many patients.

Given the frequency of incidentally discovered meningiomas, it may be reasonable to observe with clinical and radiographic follow-up in patients without symptoms. Though there is no consensus regarding the best protocol for observation, an initial interval of 6 months with subsequent annual surveillance has been proposed (50). Alternatively, patients can be reevaluated in 3 months, then 9 months, and subsequently annually (51). One limitation of observation is lack of pathologic diagnosis confirming meningioma and inability to definitively grade the lesion. Furthermore, meningioma growth can vary widely based on grade. Overall, meningiomas have been reported to grow at an average of 0.24cm per year (51) or 2% per year in a single axis, approximately 5.8% in volume (52). However, in studies where grade was subsequently obtained, grade 2 and grade 3 meningiomas grow significantly more rapidly than their benign counterparts (53). Though atypical and anaplastic may have over double the growth rate of grade 1, they may be comparable to one another (54). Besides grading, meningiomas in different locations may have different growth patterns, with one meta-analysis suggesting that skull base meningiomas grow significantly slower than other locations (55).

If significant growth of a presumed meningioma is discovered or the lesion is symptomatic at time of diagnosis, treatment is warranted if the patient is otherwise a good candidate. Currently, initial treatment modalities are limited to surgery and radiation therapy. Traditionally, the mainstay of treatment for meningiomas is surgical resection, which provides pathologic diagnosis, disease control, and typically ameliorates symptoms such as focal neurologic deficits, sequelae of elevated intracranial pressure, or seizures. The gold standard scale for meningioma resection grading, the Simpson Grade, was first described in 1957 and includes degree of resection not only of the macroscopic tumor, but also of adjacent involved dura, and any involved bone (56). Multiple grading systems have since been proposed as adjuvant treatment modalities have been developed to supplant surgery alone (57). While the Simpson grading system remains debated, more aggressive resection, when able to be safely performed, does confer improved progression-free and overall survival in patients with meningioma. While most dramatic in non-benign meningiomas, it remains significant in grade 1 pathology as well (58). Extent of resection affects progression free and overall survival in atypical meningiomas (59). Patients with atypical meningiomas have 5-year survival of 91.3% and 78.2% with gross total resection and non-gross total resection, respectively. Furthermore, patients with malignant meningioma have 5-year survival of 64.5% and 41.1% with gross total resection and non-gross total resection, respectively (60). However, meningiomas located at the skull base, where the surgical corridor is often limited or where tumor closely involves neurologic or vascular structures, precludes surgical resection of involved dura and bone or even macroscopic tumor.

Non-surgical treatment modalities, both standalone, as well as adjuvant have become increasingly utilized. The least invasive approach aside from observation is radiation alone. A recent meta-analysis found significantly higher progression-free survival in patients that underwent stereotactic radiosurgery *via* gamma knife compared to observation alone with at 5- and 10-year follow up and tumor control of 95% at 5 and 10 years. However, the included studies reported a range of complications of 8.3-39.1%, though most were temporary and either self-limited or addressed with steroids. Loss of tumor control was associated with T2 hyperintensity within the tumor, tumor size, and lack of calcification on imaging (61). However, given the lack of pathologic diagnosis, loss of tumor control may be dependent on tumor grade.

As a result of greater understanding of the risk of recurrence in non-benign meningiomas, radiation as adjuvant treatment after surgery has become increasingly investigated and there are conflicting data reported. A review of retrospective studies suggested the utility of adjuvant stereotactic radiosurgery or external beam radiotherapy in grade 3 and subtotally resected grade 2 meningiomas, which have higher risk of recurrence (62). Subsequently, the phase II trial RTOG 0539 reported 93.8% 3-year progression free survival in intermediate-risk patients with completely resected grade 2 meningiomas and recurrent grade 1 meningiomas that then underwent adjuvant radiation therapy (63) white those considered high risk; grade 3 meningiomas, subtotally resected grade 2 tumors, or recurrent grade 2 tumors; experienced a 3-year PFS of 58.8% after adjuvant therapy (64). A recent meta-analysis of 30 studies found that literature on the subject has been highly variable, with an overall improved progression-free survival without significant change in overall survival in patients that had gross total resection. However, the largest study currently in the literature is a recent single institution study of 170 patients. The authors report that use of adjuvant radiation therapy (89% of patients receiving at least 60Gy) significantly improved progression-free and overall survival in atypical meningiomas in both completely and incompletely resected tumors (65). Due to the heterogeneity of current clinical practice, an ongoing clinical trial is evaluating observation versus radiation therapy in postoperative patients who had gross total resection of an atypical meningioma (NCT03180268 Clinicaltrials.gov). Furthermore, a phase II trial investigating proton-beam radiation treatment in all meningioma grades is underway (NCT04278118) to evaluate the use of this new technology, as well as carbon ion radiotherapy in atypical meningiomas (NCT01166321).

There is no established systemic therapy that has been shown to effectively treat recurrent meningioma or to increase survival. A variety of systemic agents such as chemotherapy, immunotherapy, hormonal therapy, somatostatin analogues, and radionuclide therapy have been or are currently being studied. Nevertheless, there is no strong evidence that any these agents affect the natural history of recurrent meningioma.

## Targeted therapies for meningiomas and future directions

Targeted therapies are not currently included in the standard of care for treatment for meningiomas. Chemotherapies including hydroxyurea, temozolomide, irinotecan, and trabectedin have been investigated without clear efficacy. However, use of hormone receptor antagonistic medications, supported by the findings of these receptors in a subset of meningiomas, has been historically attempted with inconsistent results (66), at least in large part because of heterogeneity with how trials studies have been designed, results evaluated, and findings report (67). Tamoxifen, which binds the estrogen receptor, and mifepristone, which binds the progesterone receptor, have been investigated in decades prior. Unfortunately, these primarily small studies showed potential minor response in some patients, with the largest of which showing no efficacy compared to placebo (68).

NEO100, an intranasal administered purified form of perillyl alcohol, has been demonstrated in pre-clinical studies to target multiple pathogenic pathways by affecting the cyclin dependent kinase pathway in the cell cycle, endoplasmic reticulum stress, the JNK-stress response, telomerase function *via* disrupting TERT and mTOR protein complex formation, the Na/K ATPase, NOTCH, NF-kb, and TGFb, each resulting in anti-proliferative or tumoricidal properties (69). This treatment is now undergoing a phase II study in use of residual, progressive, or recurrent grade 2 and 3 meningiomas (NCT05023018).

However, as genetic and epigenetic alterations and their involved cellular pathways are identified in meningioma pathogenesis, groups are investigating more targeted therapies for these tumors in an effort to provide new clinical treatments. The VEGF pathway has been targeted due to its two-fold elevation in atypical meningiomas and ten-fold elevation in anaplastic tumors. Bevacizumab, which targets circulating VEGF has been reported in retrospective studies to increase progression-free survival (38, 39), while a phase II trial of sunitinib, a RTK inhibitor with antagonistic effect on VEGF receptor functioning demonstrated improved progression free survival in grade 2/3 meningiomas (Kaley 2015). Apatinib, which targets the VEGF receptor directly is being investigated in a phase II clinical trial in grade 2/3 meningiomas (NCT0501705).

The AKT1 mutant pathway has also been targeted in a case report of the use of AZD5363, a AKT inhibitor, in a patient with numerous AKT1 mutant meningiomas resulted in partial response followed by long-term progression free survival (70). Pre-clinical studies of cultured meningioma cell lines from NF2 found targeting of the histone deacetylase (HDAC)resulted in decreased AKT activation and decreased cellular growth, as well as, decreased tumor size in mouse models. Thereafter, AR-42 (REC-2282), a HDAC inhibitor was investigated in two small pilot studies in NF2 patients, which report partial response or stability in four patients and progression in three patients (71). As a result of these findings, a phase II/III trial of REC-2282 in NF2 patients with meningiomas, as well as sporadic meningiomas with NF2 mutations has begun (NCT05130866). AKT is within the mTOR and PI3K pathways, which are being investigated in clinical trials targeting mTOR (NCT03071874) and PI3K (NCT03631953) directly. Also, the trial NCT02523014 incorporates AKT targeting, as well as 3 other arms targeted inhibitors in tumors with mutations in SMO, NF2, and focal adhesion kinase (FAK) (**Table 3**).

**Table 3:** Current clinical trials investigating medical therapies for meningioma.

Agent	Phase	Pathology	Identifier	Status	Estimated Completion
NEO100	Phase 2	Grade 2, 3	NCT05023018	Not yet recruiting	2025
Apatinib	Phase 1/2	Grade 2, 3	NCT04501705	Recruiting	2025
REC-2282	Phase 2/3	NF2-mut,all grades	NCT05130866	Not yet recruiting	2027
Vistusertib (AZD2014)	Phase 2	Recurrent grade 2,3	NCT03071874	Active, not recruiting	2024
Alpelisib and Trametinib	Phase 1	Grade 1, 2, 3	NCT03631953	Recruiting	2022
Vismodegib, GSK2256098, Capivasertib, Abemaciclib	Phase 2	Grade 1, 2, 3	NCT02523014	Recruiting	2024
177Lu-DOTATATE	Phase 2	Grade 1, 2, 3 (DOTATATE PET pos)	NCT03971461	Recruiting	2023
177Lu-DOTA-JR11, 177Lu-DOTATOC	Phase 1/2	Grade 1, 2, 3 (DOTATATE PET pos)	NCT04997317	Recruiting	2025
Nivolumab, Ipilimumab	Phase 2	Grade 2, 3	NCT02648997	Recruiting	2023
Nivolumab with SRS, Ipilimumab	Phase 1/2	Grade 2, 3	NCT03604978	Recruiting	2022
Pembrolizumab	Phase 2	Grade 1, 2, 3	NCT03279692	Active, not recruiting	2025
Sintilimab	Phase 1/2	Grade 3	NCT04728568	Recruiting	2025
Avelumab, Proton Radiation	Phase 1b	Grade 1, 2, 3	NCT03267836	Active, not recruiting	2025

1.Robert, S.M., et al., The integrated multiomic diagnosis of sporadic meningiomas: a review of its clinical implications. J Neurooncol, 2021.

2.Youngblood, M.W., et al., Correlations between genomic subgroup and clinical features in a cohort of more than 3000 meningiomas. J Neurosurg, 2019: p. 1-10.

3.Proctor, D.T., et al., Towards Molecular Classification of Meningioma: Evolving Treatment and Diagnostic Paradigms. World Neurosurg, 2018. **119**: p. 366-373.

Given the prevalence of somatostatin receptor expression in meningiomas, octreotide and pasireotide, somatostatin receptor antagonists, have been investigated, with some reports of increased progression free survival compared to historical controls, but without evidence of partial or complete responses (72–75). However, new types of drugs may still utilize this target by another mechanism. Multiple studies are investigating the use of somatostatin antagonists with Lu177 radionucleotides that are internalized by the receptor-positive tumors cells and causes DNA damage resulting in cytotoxicity in other SSTR-positive tumors such as neuroendocrine tumors (76). These drugs, Luthera (NCT03971461) and 177Lu-DOTA-JR11 (NCT04997317) have varying levels of affinity for the sstr2 target.

Immune evasion has been a growing field of research and clinical development in a variety of solid tumors. Studies have found predominantly immunosuppressive type macrophages in AKT1 mutated meningiomas, while NF2 gene mutated tumors have high levels of immune active macrophages. Furthermore, circulating myeloid derived suppressor cells (MDSCs) are elevated in patients with meningiomas suggesting an effect on the systemic immune response and intra-tumoral MDSCs, as well as, immunosuppressive T regulatory cells (Tregs) are greater in high grade meningiomas compared to benign tumors (77). A possible target for therapies is *via* immune checkpoint pathways which are, in normal physiology, a mechanism to prevent autoimmunity by suppressing T-cell activity. TRAF-7 mutated meningiomas demonstrated elevated levels of programmed death ligand-1 (PD-L1), the major ligand for the programmed death checkpoint pathway, while PD-L2 is highly expressed in PI3K/AKT/mTOR pathway mutations and CTLA-4 was frequently expressed in PIK3CA and SMO mutated tumors. Elevated PD-L1 expression has been found in atypical and anaplastic meningiomas compared to benign (78), but a significant correlation between expression and survival has not yet been established (77).

Multiple phase I/II clinical trials are underway investigating immune modulating checkpoint inhibitors. The use of ipilimumab (NCT03604976) targeting CTLA-4, and nivolumab (NCT03604978) targeting PD-1, as well as, both medications together (NCT02648997) in conjunction with stereotactic radiosurgery in recurrent atypical and anaplastic meningiomas is currently being investigated. Other checkpoint inhibitors are also undergoing investigation as sole treatment targeting the programmed death pathway without radiation (NCT03279692), as well as, neoadjuvant treatment alone (NCT04728568) or in addition to neoadjuvant proton radiation (NCT03267836) prior to reresection.

## Conclusion

The diagnosis and treatment of meningiomas has remained a clinical challenge greatly affected by evolutions in understanding of natural history, epidemiology, pathogenesis, and treatment modalities. Advances in genetics and epigenetics have permitted further molecular classification of meningiomas as well as identification of molecular determinants of treatment response in meningioma. With the advent and refinement of novel technologies, clinically meaningful developments are emerging that may markedly revolutionize the management for these tumors in the future.

## Author contributions

AE contributed to conception of the manuscript. JL and AE contributed to the design of the manuscript. JL, SR, and GF-M contributed to data acquisition/assessment/review, creation of initial manuscript/tables, and critical review and revision of the manuscript. JA, RM, JKCL, AB-F, NT, MV, and AE contributed to critical review and revision of the manuscript. All authors contributed to the article and approved the submitted version.

## Conflict of interest

The authors declare that the research was conducted in the absence of any commercial or financial relationships that could be construed as a potential conflict of interest.

## Publisher’s note

All claims expressed in this article are solely those of the authors and do not necessarily represent those of their affiliated organizations, or those of the publisher, the editors and the reviewers. Any product that may be evaluated in this article, or claim that may be made by its manufacturer, is not guaranteed or endorsed by the publisher.
